# Combination Therapy with Trastuzumab and Niraparib: Quantifying Early Proliferative Alterations in HER2+ Breast Cancer Models

**DOI:** 10.3390/biomedicines11082090

**Published:** 2023-07-25

**Authors:** Ameer Mansur, Patrick N. Song, Yun Lu, Andrew C. Burns, Luke Sligh, Eddy S. Yang, Anna G. Sorace

**Affiliations:** 1 Department of Biomedical Engineering, The University of Alabama, Birmingham, AL 35233, USA; 2 Department of Radiology, The University of Alabama, Birmingham, AL 35233, USA; 3 Graduate Biomedical Sciences, The University of Alabama, Birmingham, AL 35233, USA; 4 Department of Radiation Oncology, University of Kentucky, Lexington, KY 40506, USA; 5 O’Neal Comprehensive Cancer Center, The University of Alabama, Birmingham 35233, AL, USA

**Keywords:** molecular imaging, patient–derived xenograft, biomarkers, targeted therapy, cellular proliferation, FLT–PET, PARP, HER2 antibody

## Abstract

HER2–targeted treatments have improved survival rates in HER2+ breast cancer patients, yet poor responsiveness remains a major clinical obstacle. Recently, HER2+ breast cancer cells, both resistant and responsive to HER2–targeted therapies, have demonstrated sensitivity to poly–(ADP–ribose) polymerase (PARP) inhibition, independent of DNA repair deficiencies. This study seeks to describe biological factors that precede cell viability changes in response to the combination of trastuzumab and PARP inhibition. Treatment response was evaluated in HER2+ and HER2– breast cancer cells. Further, we evaluated the utility of 3′–Deoxy–3′–[^18^F]–fluorothymidine positron emission tomography ([^18^F]FLT–PET) imaging for early response assessment in a HER2+ patient derived xenograft (PDX) model of breast cancer. In vitro, we observed decreased cell viability. In vivo, we observed decreased inhibition in tumor growth in combination therapies, compared to vehicle and monotherapy–treated cohorts. Early assessment of cellular proliferation corresponds to endpoint cell viability. Standard summary statistics of [^18^F]FLT uptake from PET were insensitive to early proliferative changes. Meanwhile, histogram analysis of [^18^F]FLT uptake indicated the potential translatability of imaging proliferation biomarkers. This study highlights the potential of combined trastuzumab and PARP inhibition in HER2+ breast cancer, while demonstrating a need for optimization of [^18^F]FLT–PET quantification in heterogeneous models of HER2+ breast cancer.

## 1. Introduction

Approximately 20 to 30% of all diagnosed breast cancers are characterized by an overexpression of the human epidermal growth factor receptor 2 (HER2) gene. This overexpression results in excessive production of the HER2 receptor, which plays a critical role in cell–to–cell communication via signal transduction. Studies have established a correlation between HER2–mediated signaling and apoptotic resistance, increased cellular proliferation, invasiveness, and angiogenesis [1,2,3]. Trastuzumab, a well–established monoclonal antibody treatment for HER2+ breast cancer, binds to the extracellular component of the HER2 receptor, limiting HER2 cleavage and leading to inhibition of angiogenesis, and DNA damage repair [4]. However, nearly 20% of early stage breast cancer and 70% of metastatic breast cancer will be resistant to the therapeutic effects of trastuzumab [5]. These statistics have prompted investigations into the synergistic effects of trastuzumab and other treatments, such as poly–(ADP–ribose) polymerase (PARP) inhibitors [6,7]. As these novel combination therapies are developed, there is a need to identify biomarkers for therapeutic responsiveness and better understand the temporal kinetics of the underlying biological changes.

PARP inhibitors have shown significant efficacy in treating patients with germline mutations in the *BRCA* genes in clinical trials [8,9]. These treatments exploit the genomic instability of cancer cells by inhibiting PARP, an enzyme associated with DNA single–stranded break repair [10]. Despite *BRCA* mutation status, preclinical studies have shown that single agent PARP inhibitors and a combination of PARP and HER2 inhibition are successful in inhibiting tumor growth [6,7,11]. For instance, Wielgos et al. demonstrated the sensitivity of HER2+ homologous recombination (HR) proficient trastuzumab–resistant and parental breast cancer cells to the PARP inhibitors, veliparib and niraparib [7]. One investigational hypothesis proposed by Nowsheen et al. mechanistically explains the susceptibility of HER2+ breast cancer cells to PARP inhibition is that HER2 activates the NF–kB signaling pathway, which has been shown to be dependent on PARP [11,12]. By inhibiting PARP, a decrease in HER2–dependent NF–kB activation leads to decreased proliferation and anti–HER2 resistance. Furthermore, trastuzumab’s ability to induce DNA damage and downmodulate the HR protein proliferating cell nuclear antigen (PCNA) may impair HR [13,14]. In turn, the presence of a PARP inhibitor and trastuzumab may result in synthetic lethality in HR–proficient HER2 overexpressing cells [15]. Such hypotheses and findings have prompted a multicenter clinical trial investigating the efficacy of niraparib in combination with trastuzumab for treating metastatic HER2+ breast cancer [16].

Given the substantial financial and time investment required for treatment, as well as therapy resistance, it is essential to establish accurate and consistent assessments of treatment response to better clinical decision making. Noninvasive molecular imaging offers a promising solution to the limitations of traditional methods of cellular biomarker quantification, such as serial tissue biopsies, which are invasive and, at times, clinically impractical [17,18]. For instance, Shah et al. demonstrated the capabilities of non–invasive molecular imaging of apoptosis, glucose metabolism, and cellular proliferation to correlate to tumor regression in trastuzumab–treated HER2+ xenografts, specifically apoptosis and proliferation [19]. Building upon that work, Whisenant et al. found that 3′–Deoxy–3′–[^18^F]–fluorothymidine (FLT), a positron emission tomography (PET) imaging tracer that characterizes cellular proliferation, provides early response assessment of trastuzumab–treated xenografts [17]. Clinically, the BROCADE, multi–institutional phase I study, evaluated the use of serial FLT–PET imaging as a biomarker of response to the incorporation of the PARP inhibitor veliparib to carboplatin and paclitaxel in patients with metastatic breast cancer. It was demonstrated that, among responders, a rapid decrease in FLT uptake was observed, measured through max standard uptake value (SUV) [20].

In this study, we investigated the capabilities of glucose metabolism and cellular proliferation in assessing response of HER2+ breast cancer cells to the novel combination of niraparib and trastuzumab in vitro. Evaluating these biological characteristics that have known matched PET imaging agents (glucose metabolism = FDG, proliferation = FLT), will provide an opportunity to match paired biological data with future clinical imaging translation. To further validate and contextualize these findings we leveraged a HER2+ patient derived xenograft (PDX) model, recognized for its accurate reflection of clinical heterogeneity [21,22], for biomarker assessment. Post–in vitro assessment, the translatability of the findings to the in vivo setting, was evaluated with treatment response in HER2+ cell line xenograft and the (PDX) model through FLT–PET imaging. Immunohistochemistry and FLT retention histogram analysis were performed to validate and further assess tracer uptake.

## 2. Materials and Methods

### 2.1. Cell Culture, Treatments, and In Vitro Assays

The HER2–negative MDA–MB–231 cells were maintained in Dulbecco’s Modified Eagle Medium (DMEM, Thermo Fisher Scientific, Waltham, MA, USA) supplemented with 10% Fetal Bovine Serum (FBS, Biotechne, Minneapolis, MN, USA), 1% 1 mM sodium pyruvate (Fisher Scientific, Waltham, MA, USA), and 1% 2 mM L–glutamine (Thermo Fisher Scientific, Waltham, MA, USA). The HER2–positive BT474 cells were obtained from American Type Culture Collection (ATCC) and cultured in an improved minimum essential medium (IMEM, Invitrogen, Carlsbad, CA, USA) supplemented with 10% FBS and 20 µg/mL of insulin (Thermo Fisher Scientific, Waltham, MA, USA), and the HER2–positive SKBR3 (ATCC) was maintained in RPMI (Thermo Fisher Scientific, Waltham, MA, USA) supplemented with 10% FBS. All cell lines were transfected with green fluorescent protein (GFP) in order to assess longitudinal cell viability with live cell microscopy.

Trastuzumab was obtained from MedChemExpress (MCE, Monmouth Junction, NJ, USA) and reconstituted in PBS (Fisher Scientific, Waltham, MA, USA) in vitro and saline in vivo. Niraparib (MCE, Monmouth Junction, NJ, USA) was reconstituted in dimethyl sulfoxide (DMSO, Thermo Fisher Scientific, Waltham, MA, USA) in vitro, and 10% DMSO + 90% (20% SBE–B–CD (MCE) in saline) in vivo, as directed by the manufacturer.

Response curves of HER2+ breast cancer cells were established with increasing concentrations of trastuzumab (0.1–100 µg/mL) or niraparib (0.1–100 µM). Glucose Uptake–Glo^TM^ assays (Promega, Madison, WI, USA) were utilized to determine in vitro glucose uptake 48 h after treatment initiation. Click–iT Plus EdU Cell Proliferation Imaging Kit Alexa Fluor^TM^ 555 (Thermo Fisher Scientific, Waltham, MA, USA) assays were utilized to assess in vitro cellular proliferation. Cells were allowed to adhere overnight then incubated with treatment for 48 h. Then incubated with 5–ethynyl–2′–deoxyuridine (EdU) at 10 mM for 3 h. The cells were then, fixed and permeabilized before adding the detection reagent. Fluorescent imaging (482 nm excitation; 524 nm emission) was utilized to capture baseline fluorescence immediately following treatment with either 1 µg/mL trastuzumab, 1 µM niraparib, or the combination. After 48 h post–treatment, the assays above were employed in 96–well plates with at least five samples per condition to measure the indicative biomarker and correlated to endpoint cell viability. All fluorescent imaging was acquired with the EVOS M7000 Imaging System (Thermo Fisher Scientific, Waltham, MA, USA) and bioluminescence with the IVIS Lumina III (Perkin Elmer, Waltham, MA, USA).

### 2.2. Animal Models

All animal experiments were reviewed and approved by the Institutional Animal Care and Use Committee (IACUC) at the University of Alabama at Birmingham under APN 21611. To establish the cell line–based tumor model, six–week–old female athymic nude mice (N = 16) were implanted subcutaneously with estradiol pellets at the nape of the neck. Twenty–four hours later, 10^7^ BT474 HER2+ breast cancer cells, suspended in serum–free media and 30% Matrigel (Fisher Scientific, Waltham, MA, USA), were subcutaneously implanted above the right shoulder and allowed to grow for approximately 6–7 weeks. The PDX model was established by engrafting BCM–3472 tumor pieces (50–100 mm^3^) into the third mammary fat pad of NOD.Cg–Prkdc^scid^Il2rg^tm1Wjl^ISzJ (NSG) mice (N = 45) with Matrigel supplement. Tumor measurements via caliper and mouse weight were assessed throughout the study and expressed using the formula: Tumor Volume = (4π/3) * (L1/2) * (L2/2) * ((L1 + L2)/4); where L1 and L2 are perpendicular tumor lengths. Tumors measuring 125–300 mm^3^ were selected. Mice were randomized and assigned to one of four treatment groups: vehicle, trastuzumab (4 mg/kg for BT474 model, and 10 mg/kg for BCM–3472 model), niraparib (50 mg/kg), or a combination of trastuzumab and niraparib. Intraperitoneal injections of trastuzumab were administered on day 0 and every 3 days following, while niraparib was administered daily via oral gavage. Treated groups were all normalized to the mean of the vehicle group.

### 2.3. FLT–PET Imaging and Analysis

[^18^F]–FLT was synthesized by the University of Alabama at Birmingham cyclotron facility on a GE FASTlab2 synthesizer, according to the literature procedures [23,24,25]. At each imaging timepoint, FLT was injected retro–orbitally with 3.68 ± 0.17 MBq (99.63 ± 4.56 µCi). Sixty minutes post–injection, the mice were imaged with a preclinical PET scanner (Sofie GNEXT PET/CT, Dulles, VA, USA) for 20 min, followed by an anatomical 80 kVp Bin 2 CT for reference. Regions of interest (ROI) were annotated on the anatomical data, then overlaid onto the PET data to extract SUV measurements per tumor and 20 mm^2^ of the shoulder muscle in VivoQuant (InviCRO, Boston, MA, USA). Proliferative voxels were identified as voxels with an SUV greater than or equal the SUV_mean_ of muscle plus two standard deviations.

### 2.4. Histological Assessment

On day 4, tumors were harvested, and sectioned transverse to the mouse body. The tumor samples were paraffin–embedded, sliced, and stained for H&E and Ki67. Sections were dewaxed with xylene (Fisher Scientific, Waltham, MA, USA) and rehydrated with EtOH. Citrate buffer was used for antigen retrieval. The slides were submerged in 3% H_2_O_2_ diluted in deionized water for 10 min. After a PBS wash, samples were permeabilized with 0.1% Triton 100 (Millipore Sigma, Burlington, MA, USA) in PBS. The primary antibody, anti–Ki67 (ab16667, Abcam, Cambridge, UK) (1:200) was incubated overnight. Anti–Rabbit–IgG (GTX83399, GeneTex, Irvine, CA, USA) was used as a secondary antibody and incubated for an hour. Each sample was then developed with the DAB substrate from the Vector kit (SK–4105, Vector Laboratories, Newark, CA, USA) for 40 s. High–resolution whole tumor sample images were captured with the EVOS M7000 (20×) and quantified in QuPath 0.4.0 [26,27]. Briefly, regions of interest were drawn on living tissue based on H&E staining and transferred manually to the corresponding Ki67 stained sample. The positive cell detection function was used to identify positively stained nuclei over the threshold of 0.37 and exclude cells with a cytoplasm signal intensity of higher than 0.37 to exclude folded tissue. Measurements are reported as positive detections per mm^2^ of viable tumor tissue.

### 2.5. Statistics and Code

Statistical comparisons were evaluated using the non–parametric, two–tailed, Mann–Whitney Wilcoxon Test and comparisons between histogram distributions were quantified via the Kolmogorov–Smirnov statistic with a significance level of *p* < 0.05. Histogram data is expressed in terms of fractional frequency to normalize for tumor volume. The fractional frequency is defined as the number of voxels at a certain SUV divided by the total number of voxels in the tumor. Linear regression was used to correlate biometrics. Outliers were identified and removed using the extreme studentized deviate method (Grubbs’ test). Statistics and graphs were performed and generated in GraphPad Prism (GraphPad Software, San Diego, CA, USA) with the aid of the Python 3.9.7 module ‘stats’ of the SciPy 1.7.3 library. QuPath scripts for histological processing and cell fluorescence assessment are publicly available at https://github.com/mansurlab/Tmab–Nirap–Quantification (accessed on 25 May 2023).

## 3. Results

### 3.1. Trastuzumab and Niraparib Inhibited Growth in HER2+ Breast Cancer Cell Lines

**Figure 1:**
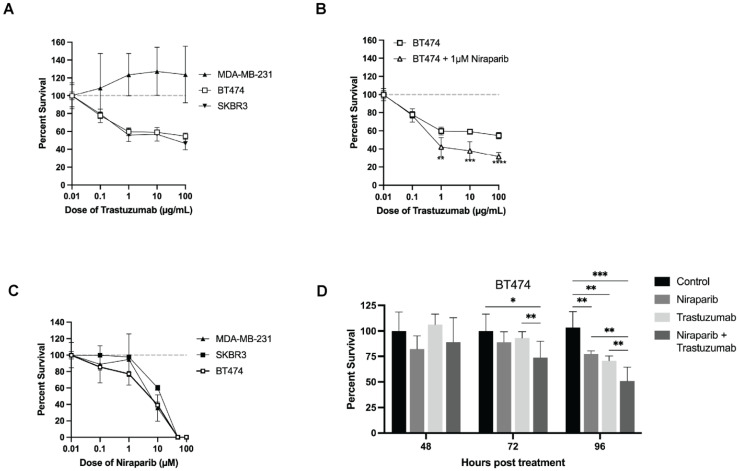
In vitro treatment response to trastuzumab and niraparib. (**A**) Trastuzumab elicited a dose–dependent response in SKBR3 and BT474 cells, whereas no response was observed in MDA–MB–231 cells. (**B**) Combining trastuzumab with 1 µM of niraparib significantly decreased survival rate in BT474 cells, compared to either monotherapy. (**C**) BT474, SKBR3, MDA–MB–231 cells exhibited sensitivity to niraparib at doses exceeding 1 µM. (**D**) No significant response is observed 48 h post–treatment but became evident at 72 h in the combination–treated group, and at 96 in all treatments. All values are reported as mean ± standard deviation. (* indicates *p* values of ≤0.05, ** *p* ≤ 0.01, *** *p* ≤ 0.001, **** *p* ≤ 0.0001).

To assess the efficacy of combining trastuzumab and niraparib treatments on HER2+ breast cancer cells, percent survival curves were established with increasing concentrations of trastuzumab (0.1–100 µg/mL) or niraparib (0.1–100 µM). In Figure 1A, the survival of HER2+ SKBR3 cells decreased to 56 ± 7.28% following treatment with 1 µg/mL of trastuzumab, meanwhile statistical differences were not observed between controls and all doses of trastuzumab in HER2– MDA–MB–231 cells. Furthermore, in HER2+ BT474 cells compared to trastuzumab alone (60 ± 4.25% survival), the inclusion of 1 µM niraparib significantly decreased percent survival to 42 ± 10.41% (*p* ≤ 0.0046), as shown in Figure 1B. All cell lines, independent of HER2 status, exhibited a high level of growth inhibition when exposed to niraparib concentrations exceeding 5 µM, as shown in Figure 1C. At 48 h post–treatment, no significant response is observed between treatment and control groups, as depicted in Figure 1D. However, response became evident at 72 h in the trastuzumab, and combination–treated groups compared to control: (control vs. trastuzumab: *p* = 0.01, control vs. combination *p* = 0.034). The trends become more apparent 96 h post–therapy in all treatments (control vs. niraparib: *p* = 0.004, control vs. trastuzumab: *p* = 0.004, and control vs. combination: *p* = 0.001). Additionally, a significant decrease in viability was observed in the combination–treated group compared to monotherapy (combination vs. niraparib: *p* = 0.0025, combination vs. trastuzumab: *p* = 0.01).

### 3.2. Cellular Proliferation Serves as a Metric of Early Response Evaluation In Vitro

**Figure 2:**
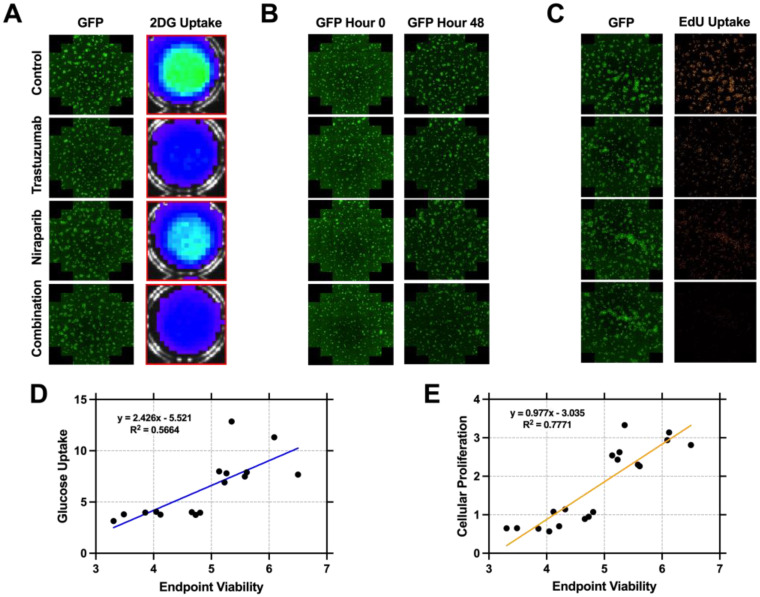
Glucose and proliferation assays of combination therapy response. Representative GFP fluorescent and 2–deoxyglucose Uptake fluorescent images taken 48 h post–treatment (**A**). GFP images from baseline and 48 h post–treatment indicate no significant changes (*p* = 0.35) in viability (**B**). Representative GFP fluorescent and 5–ethynyl–2′–deoxyuridine (EdU) uptake fluorescent images (brightness uniformly enhanced for increased figure visualization not during quantification) taken 48 h post–treatment (**C**). Each experiment condition was run at least in quintuplets. Regression analysis of glucose metabolism vs. endpoint viability (**D**) displayed an R^2^ value of 0.5664, while proliferation vs. endpoint viability (**E**) displayed an R^2^ value of 0.771. The findings indicate that proliferation has a stronger correlation to endpoint viability.

To investigate whether biomarkers of glucose uptake or cellular proliferation can inform on response prior to changes in cell viability, in vitro assays were performed simultaneously at hour 48 and correlated to endpoint cell viability. BT474 cells were treated with doses of 1 µg/mL of trastuzumab, 1 µM niraparib, or both. In Figure 2A, representative images of BT474 cells taken 48 h post–treatment are shown. The first column displays GFP fluorescent imaging, while the second shows 2–deoxyglucose (2DG) uptake bioluminescent images of the corresponding wells. Trastuzumab and combination treatment resulted in a 45.8% and 61.5% reduction of 2DG uptake compared to controls (*p* = 0.20, *p* = 0.11), while niraparib–treated wells exhibited a 23.3% decrease in 2DG uptake (*p* = 0.68) depicted in Figure S1. Figure 2B displays representative images of BT474 cells on baseline and 48 h post, no significant differences were observed in growth between treated groups and controls when normalized to baseline wells (*p* = 0.35). Figure 2C shows representative GFP and EdU (5–ethynyl–2′–deoxyuridine) uptake fluorescent images taken 48 h post–treatment. Trastuzumab, niraparib, and combination–treated wells displayed a 67.5 ± 3.27%, 22.9 ± 4.89%, and 79.7 ± 1.48% (*p* = 0.007, *p* = 0.007, *p* = 0.008) decrease in EdU uptake, respectively. Regression line analysis of the glucose metabolism (2D) and proliferation (2E) data were correlated to endpoint viability and indicate a higher correlation between proliferation and viability. Endpoint viability and glucose uptake were shown to have mild positive correlation with an R^2^ value of 0.5664 (*p* = 0.0008). Meanwhile, endpoint viability and cellular proliferation were positively correlated with an R^2^ value of 0.7771 (*p* < 0.0001).

### 3.3. Trastuzumab and Niraparib Displayed Anti–Tumoral Effects in HER2+ Xenograft and PDX HER2+ Models

**Figure 3:**
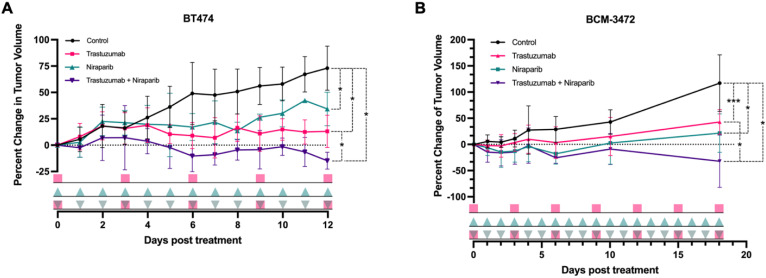
In vivo tumor response assessment. BT474 and BCM–3472 tumor–bearing mice were treated with trastuzumab, niraparib, or the combination and assessed for longitudinal changes in tumor volume as percent change from baseline (mean ± standard deviation). (**A**) Significant decreases in tumor volume were observed as early as day 5 in trastuzumab and combination–treated BT474 cohort and day 8 in the niraparib–treated BT474 cohort. (**B**) Tumor growth inhibition was not observed on day 10; however, it was subsequently observed on day 18 in treated BCM–3472 cohorts. (* *p* < 0.05, *** *p* < 0.001). The treatment dosing timelines are indicated by the three lines below the plot, with each shape indicating dose administration. The trastuzumab cohort is indicated at the top (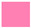 pink squares), followed by the niraparib cohort (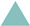 green triangles), and last, the combination cohort (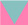 pink squares with green triangles).

To evaluate the anti–tumoral effects of trastuzumab and niraparib, longitudinal tumor volume changes were measured and are depicted as percent change from baseline for all cohorts in Figure 3. Significant inhibition of BT474 tumor growth was observed between trastuzumab, and combination–treated mice, with a mean percent change of 6.4% and −10.3% compared to vehicle 49.1% as early as day 6 (*p* = 0.016), as illustrated in Figure 3A. Meanwhile, similar significant tumor growth inhibition between the niraparib–treated tumors (13.9%) and vehicle cohort (50.8%) (*p* = 0.016) were observed after 8 days. Significant differences were observed between the monotherapy trastuzumab and combination group by day 12 (*p* = 0.028). Preliminary studies suggested that a higher dose of trastuzumab is necessary for effective treatment response, as the BCM–3472 PDX model of HER2+ breast cancer was insensitive to the 4 mg/kg dose, but responsive to doses of 10 mg/kg or greater. The dose of niraparib remained unchanged. Initial tumor regression was observed in the niraparib, and combination–treated cohorts between days 0 and 6. Compared to the vehicle group, growth inhibition was observed in all treated cohorts by day 18 (trastuzumab: *p* = 0.001; niraparib: *p* = 0.02; combination: *p* = 0.01). Endpoint mean tumor volume for each cohort was 117 ± 53.9% for control, 42.8 ± 23.5% for trastuzumab, 21.6 ± 36.7% for niraparib, and −32.2 ± 50.0% for the combination group. In both the BT474 xenografts and BCM–3472 PDX models, sustained tumor regression was only observed in the combination–treated cohort.

### 3.4. Decreasing Uptake of FLT–PET Observed in BCM–3472 PDX Model Prior to Significant Changes in Tumor Volume

**Figure 4:**
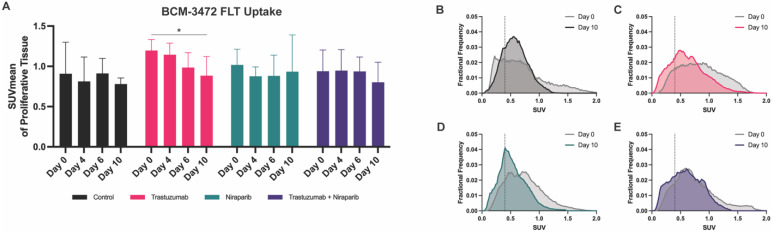
FLT uptake quantification. No significant differences in BCM–3472 tumor volume or FLT uptake were observed on days 4, 6, or 10. (**A**) Proliferative thresholding of SUV_mean_ on day 10 allowed for early identification of trastuzumab monotherapy response (*p* = 0.03). (**B**–**E**) Histogram analysis on day 10 allowed for the observation of histogram shifts in SUV compared to day 0. * *p* < 0.05.

No significant differences in tumor volume were observed on day 10 in any of the treated cohorts as indicated prior, in Figure 3B. Moreover, any changes in mean SUV from baseline to day 10 were found to be nonsignificant within groups. Using a proliferative threshold to identify uptake in viable tumor tissues, it was shown that there was a significant reduction in FLT uptake on day 10 compared to the baseline in trastuzumab–treated mice (*p* = 0.03). Fractional frequency histogram analysis on day 10 helped classify the heterogeneous samples and revealed differences in the distributions of voxel intensities compared to the baseline, as depicted in Figure 4B–E. The KS–distance values between baseline day 0 and day 10 for the control, trastuzumab, niraparib, and combination cohorts were determined to be 0.36 (*p* = 0.001), 0.22 (*p* = 0.0158), 0.22 (*p* = 0.0158), and 0.28 (*p* = 0.008), respectively, indicating significant deviations in the distributions of voxel intensities, correlated to FLT uptake, from the baseline. In addition, each cohort was compared to the controls on day 10; no significant differences were observed with the niraparib group (*p* = 0.46); however, a broader distribution was observed in the trastuzumab (*p* = 0.0008) and combination (*p* = 0.07) treated groups. Voxels categorized as necrotic, failing to meet the proliferative threshold, were observed to exhibit an SUV value of approximately 0.4.

**Figure 5:**
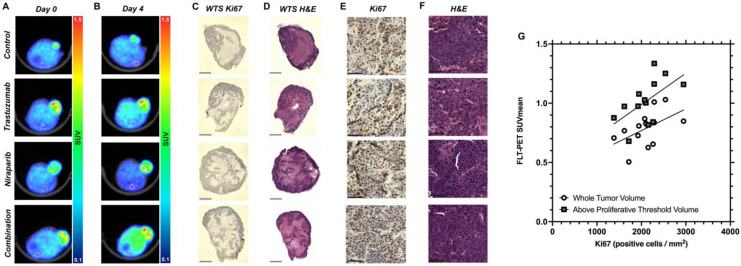
Biological validation of FLT imaging. Treated BCM–3472 tumor bearing mice were euthanized on day 4 and tumors transversely sliced. (**A**) Representative baseline FLT–PET/CT images of BCM–3472 control and treated tumors. (**B**) Representative day 4 FLT–PET/CT images of BCM–3472 control and treated tumors. Representative whole tumor sections (WTS) stained with (**C**) Ki67 immunohistochemistry (IHC) and hematoxylin counterstained, (**D**) hematoxylin and eosin. Gray bars in the bottom left depict a scale bar of 600 µm. Representative image sections of viable tissue stained with Ki67 (**E**) and H&E (**F**). Black bars in the bottom left depict a scale bar of 60 µm. (**G**) Significant correlation between Ki67 staining, quantified through positive cells per mm^2^ of viable tissue, and SUV_mean_ only after proliferative thresholding (*p* = 0.0375).

Representative baseline and day 4 FLT–PET/CT images of both control and treated BCM–3472 tumors (Figure 5A,B) are displayed. Consistent with FLT uptake observations on day 4, there were no significant alterations in proliferation quantified from IHC. Trastuzumab treatment resulted in a 3.02% reduction in proliferation compared to the control group. In contrast, niraparib–treated tumors slightly increased in proliferation by 15.57%, while the combination–treated tumors showed a 5.20% decrease in comparison to the control group. Representative histology is depicted in Figure 5C–F. Notably, a significant correlation (Figure 5G) was observed between Ki67 staining, quantified as positive cells per mm^2^ of viable tissue, and SUV_mean_ only after the proliferative threshold was implemented (*p* = 0.0375).

## 4. Discussion

In the present study, we aimed to investigate the potential of glucose metabolism and cellular proliferation as indicators for assessing the response of HER2+ breast cancer cells to the novel combination of niraparib and trastuzumab. Our observations showed that cellular proliferation, rather than glucose metabolism, emerged as a more suitable metric to assess the response of combination–targeted therapies in breast cancer. Further, we demonstrate that this combination shows therapeutic efficacy in a low HER2 expression patient–derived xenograft model. FLT–PET also provides the opportunity to quantify variation of heterogeneity of proliferation in these heterogeneous tumors, which provides insight into differential responses. Validation and further insights into tracer uptake in heterogeneous models confirmed proliferation in vitro, in vivo, and in PDX models; immunohistochemistry and FLT retention histogram analysis indicated a strong correlation between FLT and Ki67 IHC staining. Further, it was demonstrated that in necrotic tumors, it is critical to utilize other metrics of image analysis and not solely rely on mean tumor assessment through SUV to understand the biological changes from FLT–PET imaging.

In vitro findings corroborated the published literature reports on niraparib and trastuzumab responsiveness in HER2+ breast cancer [6,28]. Additionally, we discovered a direct correlation between cellular proliferation probed at hour 48 post–treatment and endpoint viability in BT474 cells. These observations align with Nowsheen’s mechanistic hypothesis that PARP inhibition leads to a decrease in HER2–dependent NF–kB activation, thereby reducing cellular proliferation. These alterations in proliferation were observed prior to changes in cell viability, signifying the marker’s role in response. Assessing proliferation may provide a better avenue to understand tumor response than [^18^F]–fluorodeoxyglucose (FDG), however, additional testing is required.

Shah et al. and Whisenant et al. have demonstrated the use of noninvasive FLT imaging to assess proliferative changes in accordance with trastuzumab response [17,19]. However, xenograft models lack several key features that would enhance clinical representation. PDX models present a superior alternative since they maintain histological and molecular characteristics of patient tumors and enable complex modeling of the heterogeneous clinical tumor microenvironment, which includes the stromal and immune cell components, which are critical for assessing tumor response interactions [21]. Furthermore, PDX models’ clinical translatability has been found to align closely with patient outcomes in chemotherapy response and was used to predict outcomes [22]. PDX models, however, pose their own set of challenges in preclinical imaging and therapy studies. Due to their aggressive nature and histological structure, they tend to develop with varying growth rates and often have highly necrotic cores accounting for 30–60% of total tumor volume, as confirmed by H&E. These factors necessitated an increased trastuzumab dosing from 4 mg/kg to 10 mg/kg to observe response (however, we note both doses are still within clinical relevance). Additionally, we observed a delayed response compared to similar–sized cell line xenografts, as seen in Figure 3. Our hypothesis initially posited that FLT uptake would decrease longitudinally in the presence of HER2–targeted treatment. However, due to the complexities of the PDX model, this trend was only observed after applying a proliferative threshold. These findings emphasize the need for more comprehensive analysis of tumoral heterogeneity, beyond just a mean value, in order to accurately capture the underlying biological changes.

Preclinical oncology studies have demonstrated a rapid decrease in FLT uptake in response to a range of anti–cancer therapies, including chemotherapies [29,30], radiotherapies [31,32], and some protein kinase inhibitors [33,34,35,36]. While this has been consistently demonstrated, Weber et al. indicate that FLT–PET changes were only partially reflective of therapeutic response when compared to FDG–PET in some of these studies indicating it might be disease– and drug–specific [37]. In addition to these factors, histological phenotypes must be addressed in heterogeneous tumors. Hence, we introduced a proliferative threshold to accommodate for necrotic cores and accurately assess changes in FLT retention in viable tissue. Furthermore, studies have shown that utilizing voxel–based histogram analysis of quantitative imaging metrics is superior to summary statistics, such as mean or max, as they were able to extract and observe longitudinal changes in biological tumor heterogeneity, such as of the tumor vascularity [38]. Such results indicate the need for further characterization of imaging data to better describe the three–dimensional tumor.

One opportunity for extension of this study is to expand the range of HER2 expression of the tumors utilized, as the scope of HER2 expression explored was restricted to HER2– and HER2+. Using strictly HER2+ and HER2–, cells may not fully capture the complexity of breast cancers, as breast tumors are known for dynamic and sometimes treatment–altered levels of HER2. The addition of a model featuring mixed HER2 expression would be beneficial. Moreover, our reliance on noninvasive molecular imaging, despite its advantages over invasive biopsies, presents its own set of challenges in heterogeneous PDX models. As observed, the living tissue proliferating around the periphery of tumors can make it difficult to accurately assess the full extent of viable tissue proliferation given the limited resolution of PET imaging. The imaging’s ability to reflect the extent of proliferation within these actively growing regions is a limitation that future work should address through multimodal imaging. Further opportunities to expand this study’s findings include the combination of niraparib with other HER2–targeted therapeutics, such as pertuzumab or small molecular tyrosine kinase inhibitors such as lapatinib.

Given these observations, it is evident that the combination of niraparib and trastuzumab presents a promising therapeutic strategy for HER2+ breast cancer. Our study demonstrates the potential use of cellular proliferation as an indicator in assessing response to the novel treatment combination. While FLT–PET has demonstrated a correlation to trastuzumab response in xenograft models [17,19], and proliferation correlates with cancer cell endpoint viability, our findings suggest that cellular proliferation, although insightful, should not be relied upon as a singular predictor of treatment response in a heterogeneous tumor population. These results emphasize the need for a multiparametric approach to capture the complexity of treatment response within these diverse and heterogeneous models.

## Data Availability

Scripts to process data and example data can be found at: https://github.com/mansurlab/Tmab–Nirap–Quantification.

## Supplementary Materials

The following supporting information can be downloaded at: https://www.mdpi.com/article/10.3390/biomedicines11082090/s1, Figure S1: Quantified glucose and proliferation assays of single and combination therapy response.
