# Supplementary material for: Combination Therapy with Trastuzumab and Niraparib: Quantifying Early Proliferative Alterations in HER2+ Breast Cancer Models
Source: Biomedicines. 2023 Jul 25;11(8):2090. doi: 10.3390/biomedicines11082090 (PMC10452700; doi:10.3390/biomedicines11082090)
Supplement: Supplementary file 1 [file biomedicines-11-02090-s001.zip › biomedicines-2455041-supplementary.pdf]

Supplemental Materials

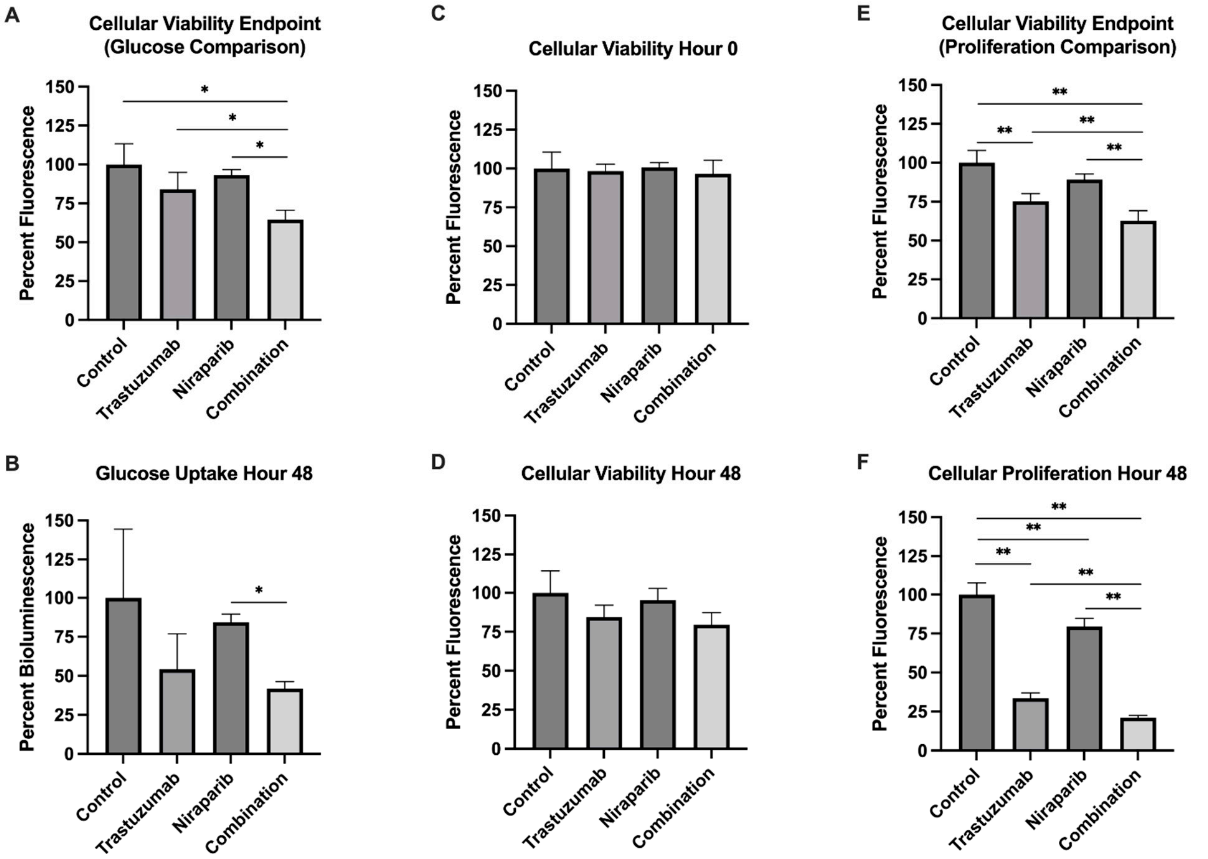

Supplemental Figure S1. Quantified glucose and proliferation assays of single and combination therapy response. The mean percent GFP fluorescence, measured 96 hours post-treatment (A) and bioluminescence quantification of 2-deoxyglucose uptake, recorded 48-hours post-treatment (B) suggest varying glucose uptake in treated groups. Baseline mean percent GFP fluorescence (C) and 48-hours post-treatment (D) denote a consistent seeding density initially, followed by slight yet insignificant decreases observed 48-hours post-treatment. The mean percent GFP fluorescence taken 96-hours post-treatment (E) and the 48-hours post-treatment fluorescence of 5-ethynyl-2'-deoxyuridine (EdU) uptake (F) signifies a decrease in proliferation in the groups treated with trastuzumab and combination therapies. All groups were normalized to mean of control for ease of comparison. (* indicates p values of ≤ 0.05, ** $p \leq 0.01$)
